# Validation of circulating histone detection by mass spectrometry for early diagnosis, prognosis, and management of critically ill septic patients

**DOI:** 10.1186/s12967-023-04197-1

**Published:** 2023-05-23

**Authors:** José Luis García-Giménez, Eva García-López, Salvador Mena-Mollá, Jesús Beltrán-García, Rebeca Osca-Verdegal, Elena Nacher-Sendra, Carmen Aguado-Velasco, Germán Casabó-Vallés, Carlos Romá-Mateo, María Rodriguez-Gimillo, Oreto Antúnez, José Ferreres, Federico V. Pallardó, Nieves Carbonell

**Affiliations:** 1 Center for Biomedical Research Network On Rare Diseases (CIBERER), Carlos III Health Institute, Valencia, Spain; 2 INCLIVA Biomedical Research Institute, Valencia, Spain; 3 Department of Physiology, Faculty of Medicine and Dentistry, University of Valencia, Valencia, Spain; 4 Department of Medicine, University of California San Diego, La Jolla, CA USA; 5 EpiDisease S.L. (Spin-Off CIBER-ISCIII), Parc Científic de la Universitat de València, Paterna, Valencia Spain; 6 Intensive Care Unit, Clinical University Hospital of Valencia (HCUV), Valencia, Spain; 7 Proteomics Unit, SCSIE-University of Valencia, Burjassot, València Spain; 8 Present Address: INCLIVA Biomedical Research Institute, Valencia, Spain

**Keywords:** Histones, Mass spectrometry, Sepsis, Septic shock, Disseminated intravascular coagulation, Diagnosis, Prognosis

## Abstract

**Background:**

As leading contributors to worldwide morbidity and mortality, sepsis and septic shock are considered a major global health concern. Proactive biomarker identification in patients with sepsis suspicion at any time remains a daunting challenge for hospitals. Despite great progress in the understanding of clinical and molecular aspects of sepsis, its definition, diagnosis, and treatment remain challenging, highlighting a need for new biomarkers with potential to improve critically ill patient management. In this study we validate a quantitative mass spectrometry method to measure circulating histone levels in plasma samples for the diagnosis and prognosis of sepsis and septic shock patients.

**Methods:**

We used the mass spectrometry technique of multiple reaction monitoring to quantify circulating histones H2B and H3 in plasma from a monocenter cohort of critically ill patients admitted to an Intensive Care Unit (ICU) and evaluated its performance for the diagnosis and prognosis of sepsis and septic shock (SS).

**Results:**

Our results highlight the potential of our test for early diagnosis of sepsis and SS. H2B levels above 121.40 ng/mL (IQR 446.70) were indicative of SS. The value of blood circulating histones to identify a subset of SS patients in a more severe stage with associated organ failure was also tested, revealing circulating levels of histones H2B above 435.61 ng/ml (IQR 2407.10) and H3 above 300.61 ng/ml (IQR 912.77) in septic shock patients with organ failure requiring invasive organ support therapies. Importantly, we found levels of H2B and H3 above 400.44 ng/mL (IQR 1335.54) and 258.25 (IQR 470.44), respectively in those patients who debut with disseminated intravascular coagulation (DIC). Finally, a receiver operating characteristic curve (ROC curve) demonstrated the prognostic value of circulating histone H3 to predict fatal outcomes and found for histone H3 an area under the curve (AUC) of 0.720 (CI 0.546–0.895) p < 0.016 on a positive test cut-off point at 486.84 ng/mL, showing a sensitivity of 66.7% and specificity of 73.9%.

**Conclusions:**

Circulating histones analyzed by MS can be used to diagnose SS and identify patients at high risk of suffering DIC and fatal outcome.

**Supplementary Information:**

The online version contains supplementary material available at 10.1186/s12967-023-04197-1.

## Background

Sepsis is defined as “a life-threatening condition that arises when the body's response to infection injures its own tissues” [1]. Incidence of sepsis is estimated at 48 million cases per year worldwide. Despite advances in therapeutic approaches and treatments applied in Intensive Care Units (ICUs), sepsis continues to be the leading cause of death from infection, responsible for more than 11 million deaths per year worldwide [2], of which nearly 2 million are newborns [3]. Furthermore, the cost of managing sepsis is very high, representing a staggering economic burden for health systems [2, 4]. Therefore, sepsis remains a major global health priority for the World Health Assembly (WHA) and World Health Organization (WHO), which adopted a resolution urging the 194 United Nations Member States to improve prevention, diagnosis, and management of sepsis [5].

Early detection of sepsis and initiation of appropriate patient management are critical to reduce length of ICU stay and increase survival. In sepsis, useful biomarkers may also provide additional information beyond what is obtained from clinical signs or scales, and can therefore help in clinical decision-making. For every hour of delayed antibiotic administration in septic shock (SS), the risk of death increases by about 7.6% [6]. Among the most dangerous examples of organ failure is disseminated intravascular coagulation (DIC), which can occur because of excessive and deregulated coagulation activation secondary to a systemic proinflammatory state, along with defective fibrinolysis and excessive thrombin formation [7]. Earlier, accurate identification of septic patients who could develop DIC may therefore help accelerate treatment against this severe complication. This complex and heterogeneous scenario for septic and SS patients requires new effective biomarkers able to solve these currently unmet clinical needs, which in turn may improve treatment decision-making and increase survival in septic patients [8, 9]. In summary, improved evaluation of candidate biomarkers is required to demonstrate their true utility in sepsis management [10].

From a pathophysiological and molecular perspective, SS is associated with an overwhelming inflammatory response, which contributes to systemic organ failure, including events such as septic myocardial dysfunction [11], DIC [7] and renal failure [12]. This highlights the importance of uncovering mechanisms which simultaneously mediate the hyperinflammatory response and contribute to systemic tissue damage, which may help to identify feasible candidates to become biomarkers not only for early diagnosis, but also for sepsis prognosis.

In this regard, the release of cellular nuclear content (mainly DNA and histones) due to extracellular trap formation (ETosis) by various blood cells (i.e., neutrophils, monocytes, macrophages, mast cells, dendritic cells, eosinophils, and basophils, among others) has been widely documented during sepsis [13–15]. Extracellular trap (ET) formation is a cell death mechanism with an important role in antimicrobial host responses [16], but it also increases levels of circulating histones [17, 18], which in turn contribute to organ injury during sepsis progression. In addition, the rupture of the cellular plasma membrane of endothelial cells [17] leads to histone release into the extracellular environment, propagating a wave of cellular injury [17, 19] which finally increases the risk of death. Therefore, the early release of histones into the blood stream just after infection through different types of ETosis, and their concomitant involvement in tissue damage, make circulating histones feasible candidates not only as early biomarkers for diagnosis, but also as biomarkers for disease progression and prognosis [20–23].

Here, drawing from a cohort of patients enrolled in a tertiary hospital ICU, we validate a method we proposed in 2017, based on targeted multiple reaction monitoring mass spectrometry (MRM-MS), to quantify circulating histones H2B and H3 in plasma [24]. Our method performed well for clinical application in early sepsis and SS diagnosis, as well as for identifying different subsets in terms of severity of sepsis and organ failure, and prediction of fatal outcome. Accordingly, we tested the value of circulating histone measurement to identify patients who debut with associated overt DIC or acute renal failure and could thus benefit from specific support therapies, thus evaluating the potential of histone levels as biomarkers for earlier evidence-based medical decision-making.

## Methods

### Patient and sample selection

All selected participants in this study were prospectively recruited at the sixteen-bed ICU of the tertiary referral Clinical University Hospital of Valencia (HCUV), Spain from February 2017 to March 2020. Participants were distributed into three different groups: ICU controls (n = 9), and septic and SS patients (n = 80; including 11 septic patients and 69 SS patients).

For the case group, we included consecutive admitted patients with a clinical diagnosis of community-acquired sepsis or SS according to the Sepsis-3 Consensus definition upon ICU admission [1]. Exclusion criteria were: (i) patients with a life expectancy under 24 h; (ii) patients outside the age range of 18–85 years; (iii) patients with an active neoplastic process; (iv) nosocomial sepsis patients who had previously received antimicrobials; (v) surgical patients. Patients in a post-cardiopulmonary resuscitation state, with suspected viral infection, who were pregnant or who did not provide consent were also excluded.

Various clinical, hemodynamic, analytical, and microbiological determinations were evaluated in every patient within the first 6 h of ICU admission. The worst values for the Acute Physiology and Chronic Health Evaluation II (APACHE II) and Sequential Organ Failure Assessment (SOFA) scores during the first 24 h were also recorded.

The control group consisted of nine subjects suffering from spontaneous haemorrhagic stroke admitted to the ICU with non-septic-related processes, applying the same exclusion criteria as for cases.

All plasma samples were stored in the INCLIVA Biobank. Experimental protocols and methods were carried out after obtaining approval from the Biomedical Research Ethics Committee (CEIm) of HCUV and performed following the relevant guidelines and regulations in accordance with the World Medical Association Declaration of Helsinki regarding ethical conduct of research involving human subjects.

### Sample preparation

Plasma samples were centrifuged for 15 min at 15,000 x*g* at room temperature for delipidation. 2 µL of each plasma sample were diluted in 8 µL H_2_O. 2µL of each 1/5 diluted plasma (approx. 2 µg) were mixed in 18 µL of 50 mM ABC(ammonium bicarbonate) together with 10 fmol of Heavy Mix peptides (SpikeTides™ TQL: LLLPGELAK for H2B, STELLIR for H3). SpikeTides™ TQL consisted of heavy-isotopically labelled proteotypic peptides that terminate with a C-terminal heavy Lys: ^13^C6,^15^N2; Arg: ^13^C6,^15^N4 (JPT Peptide Technologies, Berlin, Germany). Next, cysteine residues were reduced by 1 mM DTT (DL-Dithiothreitol) in 50 mM ABC at 300W microwave for 3 min. Sulfhydryl groups were alkylated with 5 mM IAA (iodoacetamide) in 50 mM ABC in the dark at room temperature for 20 min. Samples were subjected to trypsin digestion with 5 μg (2.5 μg/μL) of Sequencing Grade-Modified Trypsin (Promega) in 50 mM ABC at 300W microwave for 4 min. The reaction was stopped with TFA (trifluoroacetic acid) at a final concentration of 1%.

### Mass spectrometry analysis

The MRM experiments were performed in a 6500 QTRAP hybrid triple quadrupole/linear ion trap mass spectrometer (SCIEX, Framingham, MA, USA), equipped with an Ekspert nanoLC 425 nanoflow (Eksigent Technologies, SCIEX, Framingham, MA, USA) chromatographic system. 5 µL of tryptic digest (about 5 µg of protein and 10 fmol of each heavy peptide) were injected into a trap column (20 × 0.3 mm Trap Cartridge Luna® C18CL 5 µm, Phenomenex) and then loaded and separated onto an analytical column (ChromXP C18, 120 Å, 3 µm, 150 × 0.3 mm). Elution was carried out with a linear gradient of 3–40% B in A for 30 min (A: 0.1% TFA; B: ACN, 0.1% FA) at a flow rate of 5 µL/min.

The 6500 QTRAP was operated in MRM mode. MRM data were acquired in the positive mode with a spray voltage of 5500 V, curtain gas: 30psi, ion source gas: 25psi, entrance potential (EP): 10 and exit potential (EXP): 10. Collision energy (CE) and declustering potential (DP) were optimized previously for each transition.

Data analysis was conducted, area ratios (light/heavy) calculated using the Skyline 19.1.1.283 software (MacCoss lab), and light concentration estimated as fmol/µL of initial serum.

### Statistical analysis

Statistical analysis was performed with IBM SPSS 26 (IBM Corp., Armonk, N.Y., USA). Conformity of the normality of the variables in the study groups was tested using the Kolmogorov–Smirnov test. Variable values were represented as median and interquartile range for continuous variables and as percentages for discrete variables. Kruskal–Wallis test and post-hoc Dunn’s multiple comparisons test were used to compare variables between the three study groups. Two-tailed Mann–Whitney U test was used to calculate between-group statistical differences in histones levels, whereas correlations between selected groups were assessed by Spearman’s correlation tests. *P* < 0.05 was considered significantly different. ROC curves were constructed to test the performance of future in vitro diagnostic tests at ICU in septic patient management. Spearman’s Rho correlation (*r*) coefficients were calculated among the different clinical and biochemical variables and the concentration of circulating histones. The results were interpreted according to degree of association as strong (*r* = 0.7–1), moderate (*r* = 0.5–0.7), or low (*r* = − 0.5), after taking significant correlation (or) values into consideration. Graphical representations were performed using GraphPad Prism version 9.0 for MacOs X (Graph Pad Software, San Diego, CA, USA).

## Results

### Patient cohort baseline characteristics

The study was performed using a total of 89 plasma samples taken within the first 6 h after ICU admission from patients with clinical suspicion of sepsis, and subjects with spontaneous hemorrhagic stroke (used as ICU controls). Patients were classified into three different groups consisting of 9 (10%) ICU controls and 80 (90%) cases, among which 11 samples (14%) belonged to patients with sepsis diagnosis and 69 samples (76%) belonged to SS patients. It should be noted that two patients categorized as septic were undergoing vasopressor treatment, and required hemodynamic support in the form of orotracheal intubation and invasive mechanical ventilation (IMV) with intravenous sedation, instead of the SS condition (Table 1). Microbiological documentation was possible in 84% of cases. Regarding infection source, respiratory and abdominal focus predominated, and 42% of cases presented bacteriemia. In the SS group, 13 out of 69 patients (19%) finally died, of whom 12 (92%) had bacteremia (p Chi square < 0.001). In overt DIC patients’ mortality reached 30%. Clinical characteristics of the patients included in the study are shown in Table 1.

**Table 1:** Baseline clinical features of patients at ICU admission

Participant characteristics	Control N = 9	Sepsis N = 11	Septic Shock N = 69	P value
Age (years) median (IQR)	68 (30)	53 (26)	67 (16)	n.s
Male sex n (%)	4 (40)	8 (60)	41 (67)	n.s
APACHE II score median (IQR)	11 (17)	17 (8)	22 (10)	** < 0.001**^b^
SOFA score first day median (IQR)	3 (4)	4 (5)	10 (5)	** < 0.001**^b^
Charlson Index > 3 n (%)	0	3 (25)	27 (39)	n.s
Antibiotic 2 weeks prior to admission n (%)	0	1 (8)	8 (12)	n.s
Hospital admission in the previous 3 months n (%)	0	0	17 (25)	**0.048**^c^
**Infection source**				
Abdominal n (%)	–	1 (9)	23 (33)	n.s
Respiratory n (%)	–	7 (64)	21 (30)	n.s
CNS n (%)	–	1 (9)	2 (3)	n.s
Urinary n (%)	–	2 (18)	17 (25)	n.s
Catheter n (%)	–	–	1 (1)	
SSTI n (%)	–	–	3 (4)	
Unknown n (%)	–	–	2 (3)	
Bacteriemia n (%)	0	2 (18)	32 (46)	n.s
**Microorganisms**				
MDR^a^ n (%)	–	–	7 (10)	
Non-MDR^b^ n (%)	–	7 (64)	52 (75)	n.s
Unknown n (%)	–	4 (36)	9 (13)	n.s
*Candida spp.* n (%)	–	–	1 (2)	
Organ support and other therapies				
Antimicrobials within first hour n (%)	–	5 (45)	30 (45)	n.s
Corticosteroid therapy n (%)	1 (11)	2 (18)	29 (42)	n.s
Crystalloids (mL) within first 3 h	–	1500 (1625)	2000 (1000)	n.s
Vasopressor therapy n (%)	0	2 (18)	–	** < 0.001**
RRT n (%)	0	0	15 (22)	n.s
IMV n (%)	3 (33)	4 (36)	28 (41)	n.s
**Analytical data**				
White blood cell count median (IQR)	13,020 (1030)	18,000 (18,100)	12,140 (15,460)	n.s
CRP (mg/L) median (IQR)	15 (20)	285 (128)	234 (191)	** < 0.001**^a^
Procalcitonin (ng/mL) median (IQR)	0.05 (0.08)	2.90 (15.62)	31.80 (93.13)	** < 0.001**^b^
Lactate levels at first hour (mmol/l) median (IQR)	1.50 (0.55)	2.00 (0.90)	3.85 (4.55)	** < 0.001**^b^
Glucose (mg/dL) median (IQR)	125 (46)	157 (78)	135 (105)	n.s
Platelet count median (IQR)	236,000 (91,000)	208,000 (158,000)	147,000 (148,250)	**0.002**^b^
Quick index median (IQR)	91 (24)	73 (17)	61 (31)	** < 0.001**^b^
D-Dimer (ng/ml FEU) median (IQR)	581 (199)	1121 (1170)	3145 (5684)	**0.008**^b^
PT (seconds) median (IQR)	26 (8)	35 (15)	40 (18)	**0.001**^b^
**Outcomes**				
ICU LOS (days) median (IQR)	4 (6)	5 (7)	6 (11)	n.s
Hospital LOS (days) median (IQR)	21 (15)	10 (10)	16 (25)	n.s
ICU mortality n (%)	2 (22)	2 (18)	13 (19)	n.s

(−) not available. *n.s* not significant. Data are expressed as median (IQR) or n (%)

*APACHE* ^Acute Physiology and Chronic Health Evaluation; CRP C−reactive protein; ICU Intensive Care Unit; IMV Invasive Mechanical Ventilation; *LoS* length of stay; *PT* Prothrombin time; *RRT* Renal Replacement Therapy; *SSTI* Skin and Soft Tissue Infection; *SOFA* Sequential Organ Failure Assessment; *MDR* multidrug resistant microorganisms, aMDR (extended Spectrum Betalactamase−producing *Escherichia coli*, MR *Pseudomonas aeruginosa* and Methicillin Resistant *Staphylococcus aureus*), bNon−MDR (*Escherichia coli*, *Klebsiella pneumoniae*, *Enterococcus faecalis*, *Enterococcus faecium*, *Enterobacter cloacae*, *Streptococcus mitis* and *Streptococcus pneumoniae*). *LOS* length of stay^

^a^^Control vs sepsis^

^b^^Control vs Septic Shock^

^c^^sepsis vs septic shock^

### Potential early diagnostic value of circulating histones

To evaluate the potential of circulating histones as biomarkers for diagnosis of septic processes, H2B and H3 levels were compared between ICU controls and septic cases. Note that H2B is one of the prevalent histones released during NETosis [25], and H3 has been linked to organ failure and coagulopathy in septic patients [26]. Levels of both histones were quantified through MRM-MS-based assays, which quantify circulating histones in plasma samples, using standard curves with different concentrations of light peptides for H2B and H3 and stable isotopic labeled spike-in peptides [24], as described in the methods section. This approach allowed the absolute protein quantification of both circulating histones to evaluate inter-group differences.

**Fig. 1:**
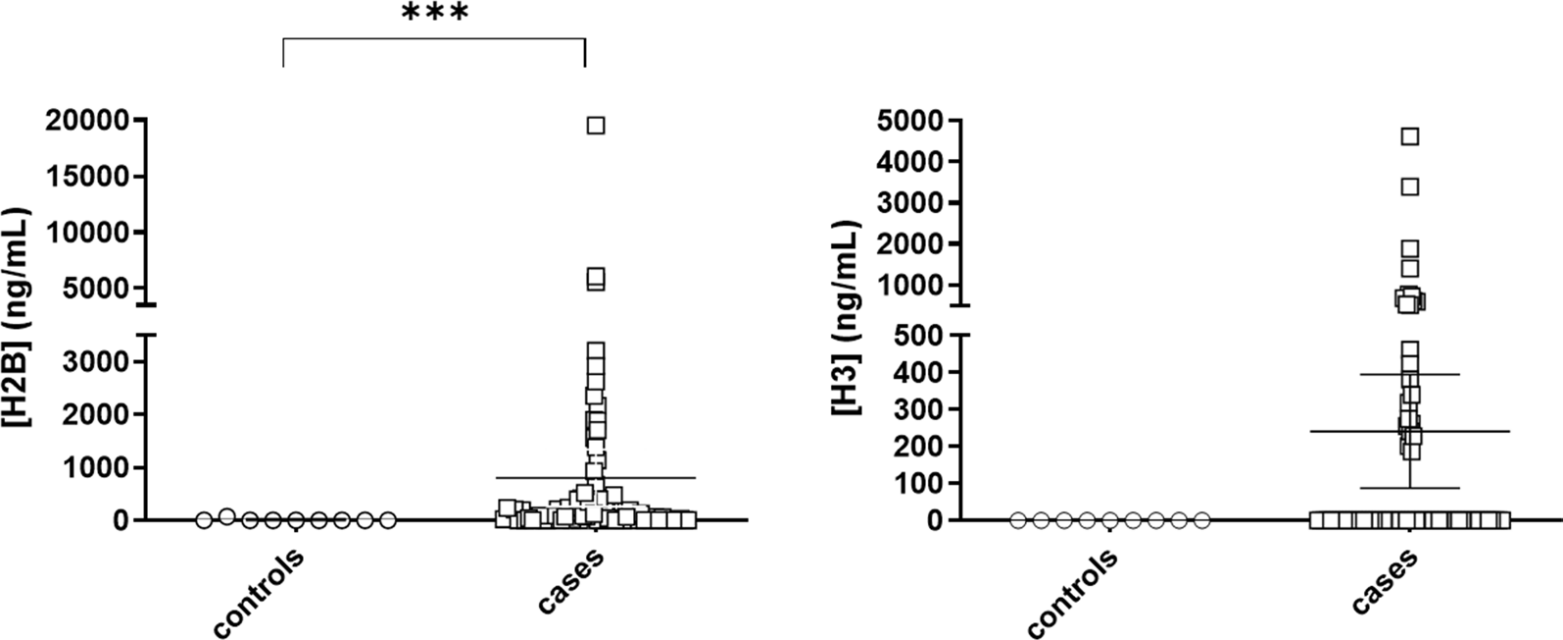
Levels of circulating histones H2B and H3 in plasma of ICU-controls and septic patients. Circulating histones were measured using LC–MS/MS using spike-in isotopic labelled peptides for absolute quantification. Groups were compared by U Mann Whitney test. *P < 0.001. The number of subjects analyzed were ICU Controls (n = 9) and cases [Sepsis + Septic Shock] (n = 80)

The median levels of circulating histone H2B found in the 9 plasma samples from ICU-controls was 0.00 ng/mL (interquartile range: 0.00), which increased up to 106.60 ng/mL (interquartile range: 423.44) in the 80 plasma samples from cases. The observed differences were statistically significant (p = 0.001 Mann–Whitney test) for histone H2B, but not histone H3 (Fig. 1).

The correlation analysis for both H2B and H3 levels revealed a Spearman´s Rho correlation index of 0.752 (p = 0.001), indicating that despite the differing absolute levels of each histone, their levels increased in parallel.

**Fig. 2:**
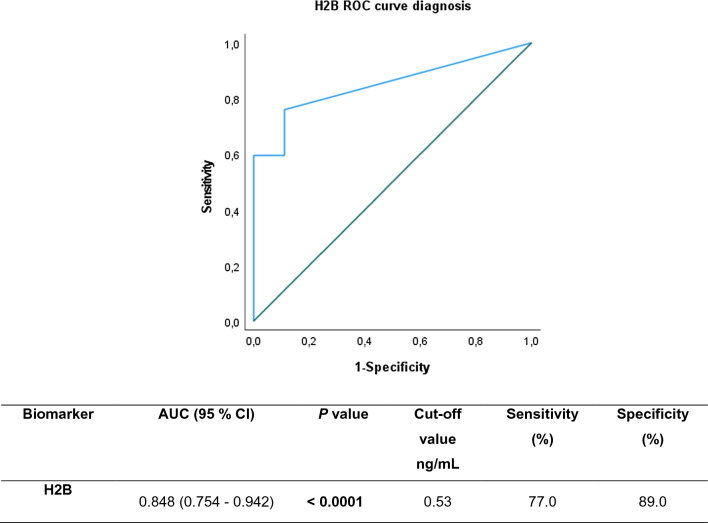
AUROC for histone H2B as a biomarker for diagnosis of septic processes. A detailed summary of the AUC, 95% CI lower and upper limit, sensitivity and specificity of the circulating histone H2B are shown in the inserted Table. The AUCs for circulating histone H2B in plasma were above 0.8, indicating that histone H2B showed high diagnostic value

Receiver Operation Characteristic curve (ROC curve) analysis also was performed to evaluate the diagnostic performance of histone H2B levels as a biomarker to distinguish septic cases from ICU-controls. The AUC (area under ROC curve), confidence interval (CI), optimal concentration cut-off value and sensitivity and specificity for histone H2B are shown in Fig. 2. The positive predictive value (PPV) of the diagnostic test based on a positive test cut-off point at 0.53 ng/mL was 98%, and the negative predictive value (NPV) was 30%.

**Table:** 

Biomarker	AUC (95% CI)	*P* value	Cut-off value ng/mL	Sensitivity (%)	Specificity (%)
H2B	0.848 (0.754—0.942)	** < 0.0001**	0.53	77.0	89.0

### Circulating histones H2B and H3 are early diagnostic predictors of septic shock

Next, we compared the ability of circulating histone levels to differentiate patient severity in an ICU setting.

In patients with sepsis, H2B median concentration levels were 22.40 ng/mL (interquartile range: 176.90). However, levels were much higher in the SS group, with a median concentration of 121.40 ng/mL (interquartile range: 446.70) [p = 0.001].

**Fig. 3:**
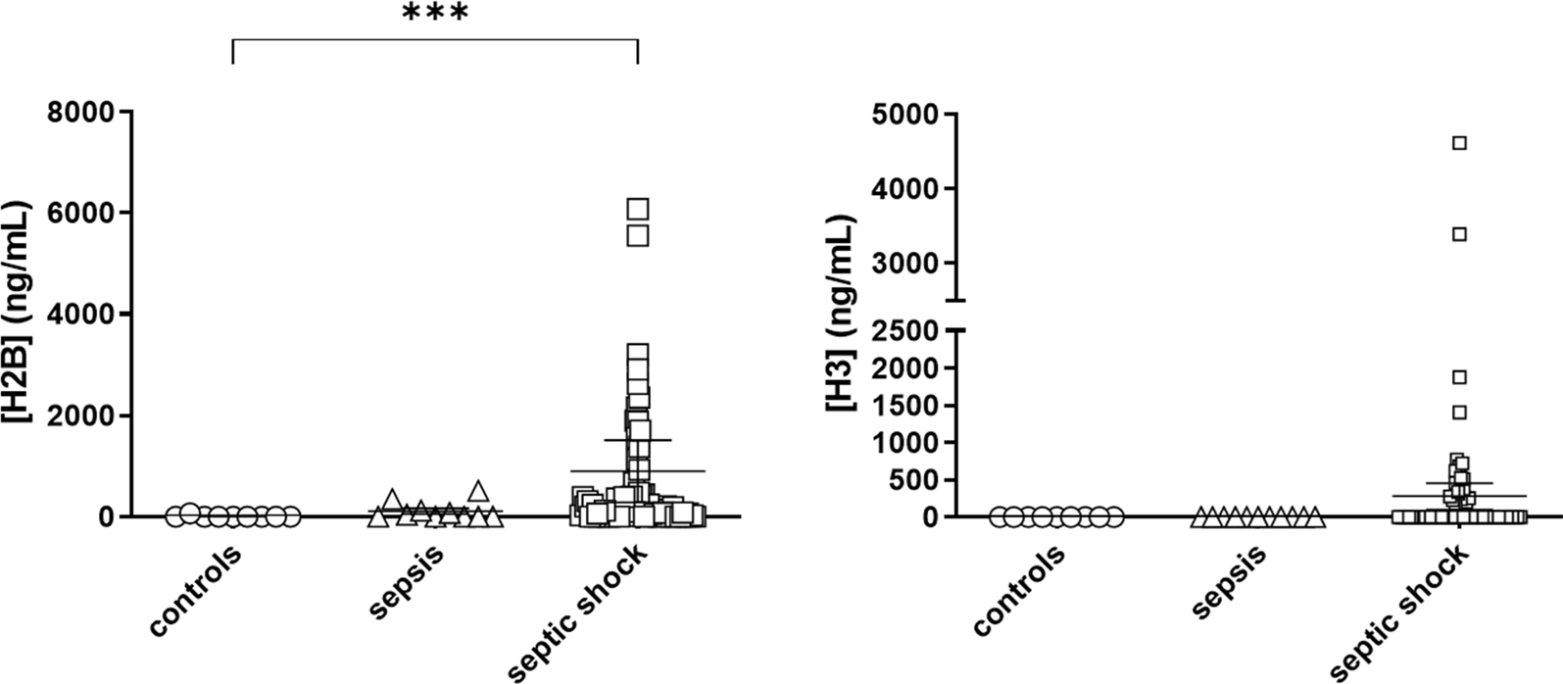
Concentration of circulating histones H2B and H3 in plasma of ICU-controls, sepsis, and septic shock patients. Circulating histones were measured using LC–MS/MS using spike-in isotopic labelled peptides for absolute quantification. Groups were compared by the Kruskal–Wallis test. *P < 0.01. The number of subjects analyzed were ICU Controls (n = 9); Sepsis (n = 11); septic shock (SS) (n = 69)

For circulating histone H3, no statistical differences were found due to the number of samples with a concentration around 0.00 ng/mL in the different groups (Fig. 3).

**Fig. 4:**
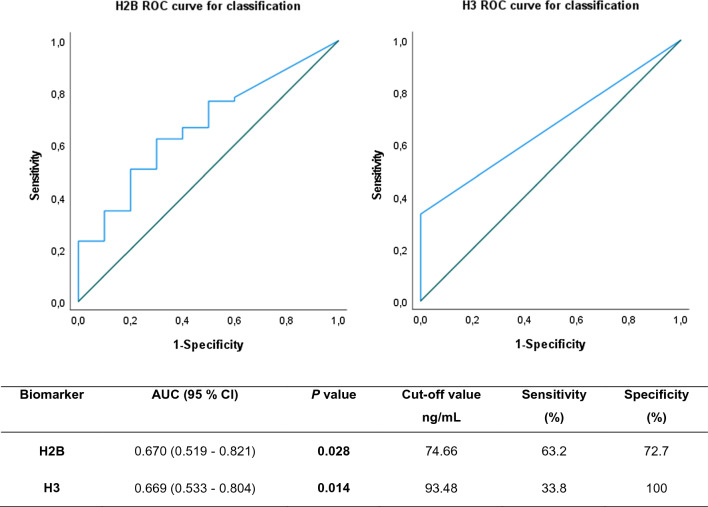
AUROCs for histones H2B and H3 levels as biomarkers for classification of septic processes (sepsis vs. septic shock). A detailed summary of the AUC, 95% CI lower and upper limit, sensitivity and specificity of the circulating histones H2B and H3 are shown in the inserted Table. The AUCs for circulating histones H2B and H3 in plasma were above 0.6, indicating that histones H2B and H3 showed a moderate classification power

ROC curve analysis was performed to evaluate the diagnostic performance obtained by measuring the concentration of histones H2B and H3 as relevant biomarkers for patients suffering from sepsis or SS classification. The results obtained from curve analysis are shown in Fig. 4.

**Table:** 

Biomarker	AUC (95% CI)	*P* value	Cut-off value ng/mL	Sensitivity (%)	Specificity (%)
H2B	0.670 (0.519–0.821)	**0.028**	74.66	63.2	72.7
H3	0.669 (0.533–0.804)	**0.014**	93.48	33.8	100

For histone H2B, the PPV of the diagnostic test based on a positive test cut-off point of 74.66 ng/mL was 92.50%, and the NPV was 24.24%. For histone H3 the PPV of the diagnostic test based on a positive test cut-off point for 93.48 ng/mL was 100%, and the NPV was 21.05%.

### Circulating histones correlate with sepsis severity-related clinical and biological parameters

To assess the relationship between circulating histones and different parameters of disease severity, we performed a Spearman’s correlation study between circulating histones H2B and H3 and different clinical variables and biochemical parameters in septic cases. First, as mentioned earlier, a strong correlation was found with both histones H2B and H3 (Spearman’s ρ = 0.771, p < 1·10^–16^). Our results demonstrated moderate, positive and statistically significant correlation in septic patients between lactate levels in the first hour after ICU admission and H2B and H3 levels (Spearman’s ρ = 0.410, p < 0.001; Spearman’s ρ = 0.509, p < 0.001, respectively).

Regarding organ failure, a significant finding was a correlation between H2B and H3 histone levels and SOFA score at ICU admission (Spearman’s ρ = 0.378, p < 0.001; Spearman’s ρ = 0.491, p < 0.001, respectively). Moreover, circulating histones showed a strong correlation specifically with coagulation-related parameters: H2B (Spearman’s ρ = 0.725, p < 0.001) and H3 (Spearman’s ρ = 0.541, p < 0.001) correlated positively with D-dimer. H2B (Spearman’s ρ = − 0.269, p = 0.017) and H3 (Spearman’s ρ = -0.285, p = 0.011) were negatively correlated with platelet plasmatic count (Additional file 1: Tables S1 and S2). Finally, histone H3 showed a moderate negative correlation with functional protein C (Spearman’s ρ = − 0.477, p = 0.001).

**Fig. 5:**
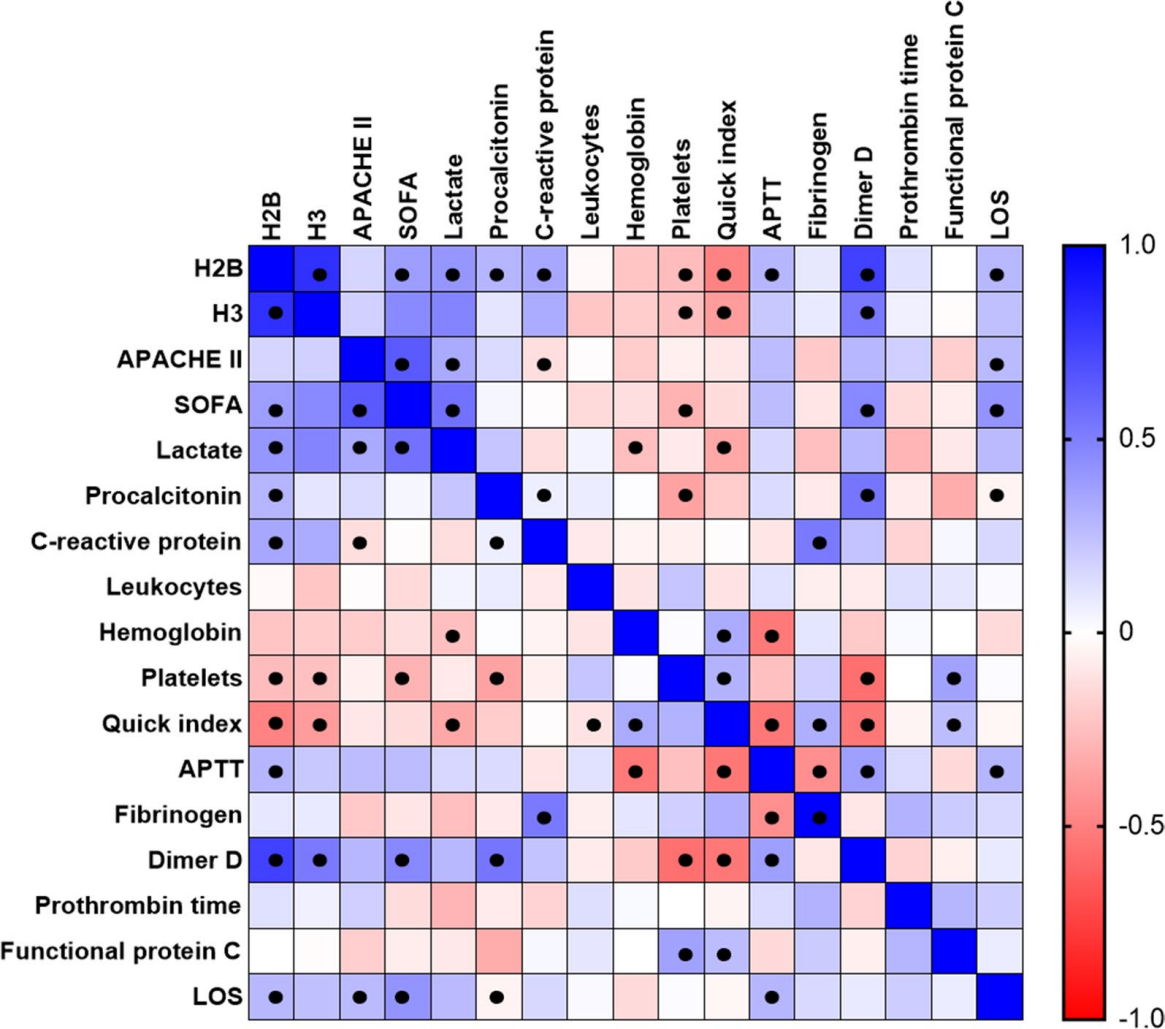
Heatmap representing the Spearman correlations coefficients (− 1 to + 1) among the clinical and analytical variables measured in septic shock cases. Red indicates negative and blue positive correlation between compared parameters. APACHE II: Acute Physiology and Chronic Health disease Classification System II; SOFA: Sequential [Sepsis-related] Organ Failure Assessment; APTT: activated partial thromboplastin time. The number of subjects analyzed were 80 septic patients (11 sepsis, 69 SS)

In addition, we performed a correlation analysis focused on SS patients (Fig. 5), finding a strong correlation in this subgroup with both H2B and H3 (Spearman’s ρ = 0.812, p < 0.001). In addition, we found significant positive correlations of histones H2B and H3 with lactate (Spearman’s ρ = 0.409, p < 0.001; Spearman’s ρ = 0.475, p < 0.001, respectively), and H3 with SOFA (Spearman’s ρ = 0.466, p < 0.001). Regarding the correlations of circulating histones with coagulation parameters, we found that in SS patients H2B and H3 showed a strong positive correlation with D-dimer (Spearman’s ρ = 0.742, p < 0.001; Spearman’s ρ = 0.519, p < 0.001, respectively), whereas both histones correlated negatively with the Quick index (Spearman’s ρ = -0.485, p < 0.001; Spearman’s ρ = − 0.385, p < 0.001, respectively). Interestingly, H2B and H3 also correlated positively with LoS (Spearman’s ρ = 0.298, p = 0.014; Spearman’s ρ = 0.315, p = 0.009, respectively). Detailed information of correlations among the different parameters analyzed can be seen in Additional file 1: Tables S1 and S2.

Afterwards, we evaluated the correlation of circulating histones H2B and H3 with PCT and PCR, currently widely used as biomarkers in sepsis. In septic cases, we found significant mild correlations between histone H2B and PCT (Spearman’s ρ = 0.319, p = 0.004), while H3 showed a negative moderate correlation with PCR (Spearman’s ρ = 0.477, p < 0.001). In SS cases (Fig. 5), we found only mild positive correlations between histone H2B and PCT and PCR (Spearman’s ρ = 0.286, p = 0.002; Spearman’s ρ = 0.342, p = 0.005, respectively), and H3 showed a moderate positive correlation with PCR (Spearman’s ρ = 0.329, p = 0.006). Black dots indicate statistical significance p < 0.05.  

In summary, our correlation analysis shows that levels of circulating histones H2B and H3 correlate with several classic biomarkers of sepsis progression, as well as with biochemical and clinical parameters related to specific processes associated with clinical management of septic patients, such as coagulation and organ failure. We therefore sought to determine whether levels of circulating histones could be used in the management of specific septic patient subsets.

### Circulating levels of histones H2B and H3 were higher in septic shock patients with organ failure requiring invasive organ support therapies

To further evaluate the potential use of histones as early biomarkers for organ failure assessment, as well as their capability to predict the need of organ support therapies within the following 24 h, we analyzed H2B and H3 histone levels in SS patients requiring organ support therapy, specifically under RRT and IMV.

**Fig. 6:**
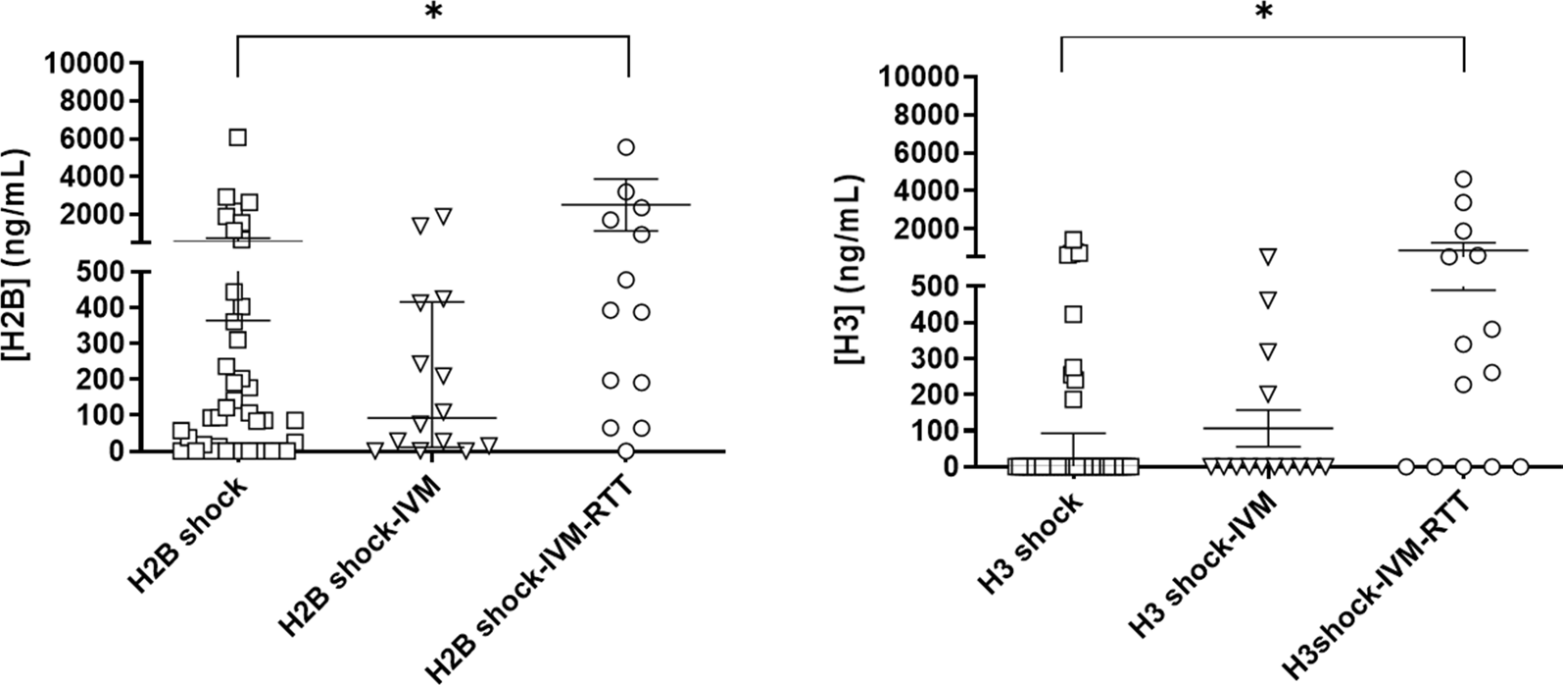
Levels of circulating histones H2B and H3 in plasma of septic shock patients with and without support therapies. Circulating histones were measured using LC–MS/MS with spike-in isotopic labelled peptides for absolute quantification. Groups were compared via Kruskal–Wallis test. *P < 0.001. The number of subjects analyzed were septic shock cases without vital support therapy (n = 41); septic shock cases with IVM (n = 14) and septic shock cases with IVM + RTT (n = 14)

A total of 15 (22%) of 69 SS patients required RRT, 28 (40%) needed IMV support and 14 (20%) received both (RRT plus IMV). The median level of histone H2B in patients with RRT was 393.19 ng/mL (interquartile range, 2289.37) vs. 93.92 ng/mL in patients who did not require RRT support [interquartile range 404.73] (p = 0.030, Mann–Whitney test). For histone H3, in patients with RRT the median concentration was 261.36 ng/mL (interquartile range, 590.08) vs. 0.00 ng/mL (interquartile range, 190.54) in patients without (p = 0.008, Mann–Whitney test). For patients requiring early IMV support, differences in histone levels were observed but were not statistically significant (Fig. 6). In patients receiving both RRT + IMV support, H2B and H3 concentrations were 435.61 ng/ml (IR 2407.10) and 300.61 ng/ml (IR 912.77), respectively (p = 0.050), compared to 93.92 ng/mL of H2B and 0.00 ng/mL of H3 in patients not requiring any organ support therapy.

### High levels of circulating histones are associated with overt DIC in septic shock patients

To evaluate the possible relationship between levels of histones H2B and H3 in septic processes and the presence of DIC, we proceeded to construct a new variable using the ISTH DIC scoring algorithm [27]. This score requires analytical parameters that individually give insufficient information to assess coagulation state as dynamic organ failure. Identifying a feasible DIC-related biomarker may therefore provide useful information to evaluate the coagulation state of critically ill patients in an early stage at ICU admittance and indicate overt DIC severity. This enables us to identify a subset of SS patients who could potentially benefit from specific management. Among the 80 samples from septic subjects (SS = 86%) only 10/69 (14.5%) SS cases were classified as overt DIC (ISTH scale ≥ 5), so values of circulating H2B and H3 were restricted to the SS group (Table 2).

**Table 2:** Values of biomarkers in septic shock patients according to ISTH Diagnostic Scoring System

Biomarker	Overt DIC (n = 10)	Non-overt DIC (n = 59)	*P* value
[H2B] ng/mL	400.44 (1335.54)	93.30 (424.47)	**0.040**
[H3] ng/mL	258.25 (470.44)	0.00 (0.00)	**0.034**

Values are expressed as median values (interquartile range)

**Fig. 7:**
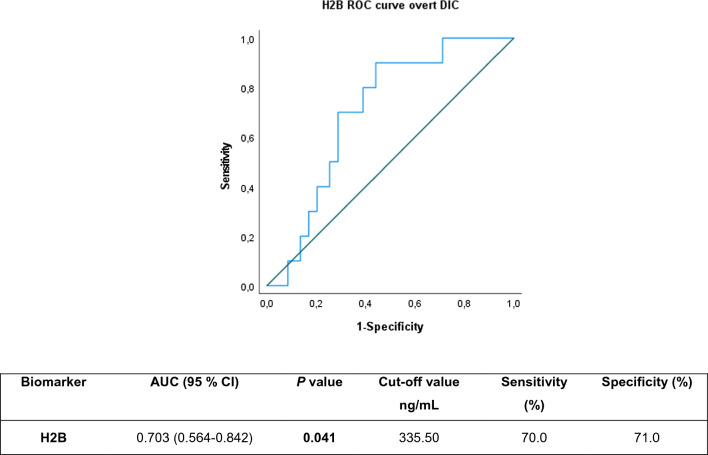
AUROC for histone H2B a as biomarker for DIC identification in septic shock patients. A detailed summary of the AUC, 95% CI lower and upper limit, sensitivity and specificity of circulating histones H2B and H3 are shown in the inserted Table. The AUCs for circulating histones H2B and H3 in plasma were above 0.7, indicating moderate diagnostic power for histone H2B

To evaluate the diagnostic performance of histones H2B and H3 in SS patients who were admitted to ICU with associated overt DIC, a ROC curve was constructed and analyzed. The ROC curve obtained was not significant for histone H3, yet we were able to identify a cut-off for H2B with a good sensitivity and specificity percentage, even higher than the threshold described above for other associated organ failures in the SS population (Fig. 7). It should be noted that H2B was measured immediately after patient ICU admission, a time when coagulation might either still not be reflected by common analytical parameters, or be in the so called pre-overt or non-overt DIC state, with the possibility to progress or not to overt DIC.

**Table:** 

Biomarker	AUC (95% CI)	*P* value	Cut-off value ng/mL	Sensitivity (%)	Specificity (%)
H2B	0.703 (0.564–0.842)	**0.041**	335.50	70.0	71.0

### Outcome prediction in septic shock patients based on circulating H2B and H3 levels

To assess the potential of histones for early prediction of fatal outcome in SS patients, we compared their values in cases diagnosed as SS with fatal outcome during the ICU stay (n = 13; 19%) and cases of SS who survived the SS episode (n = 56; 81%). For histone H2B the concentration in plasma samples from SS-surviving patients was tenfold lower than in those who died. The results obtained are shown in Table 3. The mean value for histone H3 concentration was under 163 ng/mL in all survivors, demonstrating that H3 presence in patient plasma was associated with fatal outcome in SS cases.

**Table 3:** Values of septic shock patient biomarkers according to outcome

Biomarker	Septic shock survivors	Septic shock non-survivors	*P* value
[H2B] ng/mL	93.31 (397.57)	914.20 (2799.10)	**0.008**
[H3] ng/mL	0.00 (221.10)	370.10 (1203.00)	**0.004**

Values are expressed as median values (interquartile range)

**Fig. 8:**
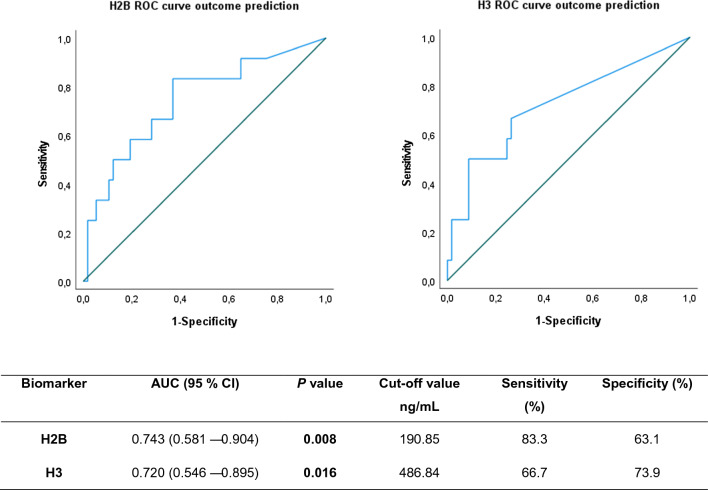
AUROCs for histones H2B and H3 as biomarkers of outcome prognosis in cases of septic shock. A detailed summary of the AUC, 95% CI lower and upper limit, sensitivity and specificity of circulating histones H2B and H3 are shown in the inserted Table. The AUCs for circulating histones H2B and H3 in plasma were above 0.7, indicating that histone H2B and H3 showed moderate classification power

The potential use of these biomarkers as outcome predictors in SS patients was assessed by a ROC curve analysis (Fig. 8). Accordingly, we found a 32% PPV of the diagnostic test based on a positive test cut-off point at 190.85 ng/mL and a 95% NPV for the histone H2B. Similarly, we obtained a 54.5% PPV of the diagnostic test based on a positive test cut-off point at 486.84 ng/mL and 89.5% NPV for the histone H3.

**Table:** 

Biomarker	AUC (95% CI)	*P* value	Cut-off value ng/mL	Sensitivity (%)	Specificity (%)
H2B	0.743 (0.581–0.904)	**0.008**	190.85	83.3	63.1
H3	0.720 (0.546–0.895)	**0.016**	486.84	66.7	73.9

**Fig. 9:**
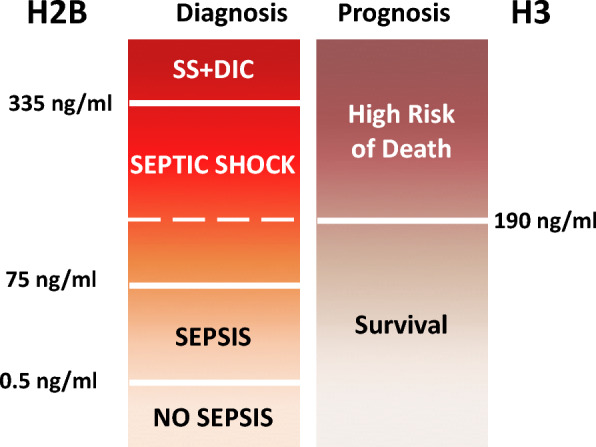
Decision thresholds according to levels of circulating histones H2B and H3 in plasma for early management of patients with sepsis. Values below 190 ng/mL for H2B are associated with increased survival probability. This result is reinforced if patients showed a value below 163 ng/mL of circulating histone H3. However, values higher than 190 ng/mL of H2B are associated with high risk of renal failure in septic shock patients and predictive of fatal outcome. When the levels of circulating histone H2B were higher than 335.50 ng/mL, there was an increased risk of developing DIC in patients. In line with this idea, we found that values for histone H2B above 435.61 ng/ml were indicative of need for vital support therapies

In summary, our results indicate that circulating histone concentration increases according to patient severity. Figure 9 represent a schema showing how levels of circulating histones can contribute to diagnosis and prognosis, classify patients requiring vital support therapies, identify pre-DIC patients, and even predict fatal outcome. Therefore, circulating histones may help physicians in deciding on the best therapeutic options and management for septic patients.

## Discussion

Cell nuclear content release as part of the inflammatory ETosis response to infection in the human organism increases levels of plasma circulating histones, which in turn contribute to organ injury during sepsis progression [17, 18]. In addition, cell rupture (during apoptosis and necrosis) in different injured tissues may substantially increase the levels of circulating histones, thus propagating a wave of cellular damage, and provoking patient death [17, 19].

Extracellular histones are responsible for cell damage via several transduction pathways [15, 20, 28–31]. It has been demonstrated that circulating histones mediate innate immunity dysregulation, triggering inflammatory responses, endothelium injury, and coagulation [28, 32–38], thus their levels are associated with sepsis severity, tissue damage and risk of death [17, 17, 21, 21–23, 39, 40]. This last mortal event is influenced mainly by endothelium damage and vascular endothelial cell death, which may in turn induce barrier function impairment, decreased blood flow, vascular tone and blood pressure, and activation of the coagulation cascade. Moreover, endothelium dysfunction affects leucocyte recruitment and activation, and contributes to the release of cytokines and other reactants which synergistically contribute to sepsis severity [41, 42].

The present study was performed to evaluate the potential use of a MS-based method to quantitatively measure circulating histones H2B and H3 [24] with the aim of rapidly identifying septic and SS patients and for predicting patient outcome in an ICU environment. Our results confirm that higher H2B and H3 blood levels correlated with the most severe stage of the septic process. Levels of circulating H2B and H3 were quantitatively higher in SS cases with organ failure requiring organ support therapy, especially in those requiring more than one therapy, those who needed a combination of RRT plus IMV, as well as in SS patients with DIC. These data suggest that measuring the levels of circulating histones can help predict potential organ failure scenarios and prompt clinical decision-making to start specific support therapies earlier. These results also agree with previous reports confirming that plasmatic histone levels are associated with sepsis-related organ dysfunction [23, 24, 30, 43]. Moreover, our data showed that early measurement of higher levels of circulating histones could predict fatal outcome in SS patients, especially by identifying a cut-off for ruling out the possibility of death. In particular, presence of H3 in patient plasma was associated with fatal outcome in SS cases, in agreement with results obtained by Yokoyama et al. [26].

Importantly, our results indicate that circulating histones concentration can further improve the management of critically ill patients in the ICU just after admission, as histone H2B was able to predict organ failure with good sensitivity and specificity among more severe SS patients. A four-fold higher mean H2B concentration was detected in SS with comorbid organ failure. We propose that the physiopathological process of SS could at least partly account for the link between circulating histone levels and endothelial damage, which is commonly present in most affected organs (i.e., circulatory system, endothelium, kidney and lungs). This finding is of special relevance given the lack of a gold standard to diagnose SS-induced DIC [44]. In fact, simple analytical data about coagulation state are currently used to diagnose sepsis-induced DIC, but scoring systems are not user friendly at bedside and physicians are often unfamiliar with them [45]. Moreover, available laboratory parameters have failed to diagnose early asymptomatic DIC, also called “pre-DIC” or “non-overt” stages [7]. In our study, H2B was useful to identify DIC in SS patients within the first hours after ICU admission, even when commonly requested coagulation parameters did not clearly define a DIC state. Our findings reinforce the notion that circulating histones contribute to DIC by damaging endothelial cells [32, 36, 46, 47]; first, by affecting extrinsic and common coagulation pathways, as demonstrated by the positive correlation between histones, DD and prolonged prothrombin time [28]; and second, due to the negative correlation found between circulating histones and platelet counts. This result is in line with previous findings in which histones could stimulate ultra-large von Willebrand factor multimers from endothelium, thus contributing to platelet activation and consumption and microvascular thrombosis [46–48] or via an alternative mechanism described recently by Abrams et al., in which histones mediate active thrombin release [43]. Furthermore, physiological anticoagulants thrombomodulin, antithrombin and activated protein C (APC) are often impaired in DIC, and these have been shown to have prognostic significance [27], probably because histones dampen thrombomodulin-dependent protein C activation, avoiding the cytoprotective functions of APC and thus enhancing plasma thrombin generation [49, 50]. This result is especially relevant because APC attenuation contributes to thrombin production, and during this process histones can also modulate APC action, which in turn reduces thrombomodulin levels [49, 51].

Importantly, circulating histones can be used to evaluate the effectiveness of current and future therapies based on their removal from the blood stream [52]. Such is the case of the use of heparins, non-anticoagulant heparins and heparinoids, monoclonal antibodies against histones or nucleosomes [53], molecules with the ability to capture circulating histones such as polysialic acid [54, 55] and small polyanions [56], and even new polymer hydrogel sequestrants [57]. The fact that circulating histones correlate negatively with APC in SS cases revives the notion of reintroducing into the market the withdrawn recombinant human APC or analogues, with the aim of providing a new battery of therapeutic strategies designed to combat sepsis and its associated morbidity and mortality.

## Limitations of the study

This is the first study validating the potential use of circulating histones using a MS method for early diagnosis and prognosis of community sepsis and SS, but this study was performed only in a limited number of patients from a single tertiary hospital. Moreover, histone measurements were performed in samples taken after patient admission in the ICU. It would be interesting to evaluate histone levels along the course of patient ICU stay to explore changes in histone levels according to sepsis progression, and observe the effect of therapies in decreasing or removing circulating histones. Another limitation of the study is the small patient sample size, due to which disaggregation into different SS patient subtypes limited the number of patients in each subgroup. Despite these suboptimal circumstances, statistical significances were nonetheless found.

## Conclusion

Early diagnostic and prognostic of sepsis is an unmet clinical need to improve patient management. Limitations regarding appropriate biomarkers have also impacted on the success of clinical trials, and the range of therapies to treat sepsis is currently lacking. In this regard, identifying biomarkers contributing to tissue damage and sepsis-associated complications may help researchers develop more effective treatments. In our study, the use of circulating histones as biomarkers suggests a potential application for early diagnosis and prognosis of septic patients. Moreover, circulating histone levels can be used distinguish between organ dysfunction and organ failure at a very early stage, permitting the use of a more aggressive strategy via earlier initiation of organ support, which in turn could help improve sepsis survival. The potential of H2B and H3 as biomarkers warrants consideration in larger cohorts of sepsis and SS patients in future studies.

## Supplementary Information

**Additional file 1:** **Table S1.** Sepsis and septic shock cases correlation analysis. **Table S2.** Septic shock cases correlation analysis.

## Data Availability

Data are treated and protected in accordance with Organic Law 3/2018 on the protection of personal data, and INCLIVA’s Privacy and Information and Security Policy. Data will be available for research upon request after approval by a INCLIVA’s Ethics Committee according to the law.

## Abbreviations

APC: Activated protein C
APACHE II: Acute physiology and chronic health evaluation II
AUC: Area under ROC curve
PaO2: Partial pressure of arterial oxygen to fractional inspired oxygen
CRP: C-reactive protein
CE: Collision energy
CI: Confidence interval
DP: Declustering potential
DIC: Disseminated intravascular coagulation
EP: Entrance potential
EXP: Exit potential
ETs: Extracellular traps
ICU: Intensive Care Unit
ISTH: International Society on Thrombosis and Hemostasis
LoS: Length of stay
METs: Macrophage extracellular traps
MDR: Multidrug resistant microorganisms
MRM-MS: Multiple reaction monitoring targeted mass spectrometry
NPV: Negative predictive value
NETs: Neutrophil extracellular traps
PTT: Partial time of thromboplastin
PPV: Positive predictive value
RRT: Renal replacement therapy
SOFA: Sequential organ failure assessment
SSTI: Skin and soft tissue infection
SD: Standard deviation
