# Supplementary material for: Validation of circulating histone detection by mass spectrometry for early diagnosis, prognosis, and management of critically ill septic patients
Source: J Transl Med. 2023 May 23;21:344. doi: 10.1186/s12967-023-04197-1 (PMC10204297; doi:10.1186/s12967-023-04197-1)
Supplement: Supplementary file 1 — Additional file 1: Table S1. Sepsis and septic shock cases correlation analysis. Table S2. Septic shock cases correlation analysis. [file 12967_2023_4197_MOESM1_ESM.pdf]

Table 1. Sepsis and septic shock cases correlation analysis

Variable		H2B	H3	APACHE II	SOFA score	C-reactive protein	Procalcitonin	Lactate	Functional protein C	Leukocytes	Hemoglobin	Platelets	Quick index	Activated Partial Thromboplastin Time	Fibrinogen	Dimer-D	Prothrombin time	ICU Lenght of stay
**H2B**	**Spearman's Correlation coefficient**	1,0000	,771**	0,1908	,378**	,292**	,319**	,410**	-0,2719	-0,0079	-0,2073	-,269*	-,446**	0,2399	0,1322	,725**	0,1198	0301*
	**Significance**		<0,0001	0,0988	0,0008	0,0094	0,0044	<0,0001	0,0708	0,9455	0,0686	0,0171	<0,0001	0,0723	0,3923	<0,0001	0,4801	0,0070
**H3**	**Spearman's Correlation coefficient**	,771**	1,0000	,229*	,491**	,276*	0,1829	,509**	-,477**	-,226*	-0,1854	-,285*	-,411**	0,2344	0,0424	,541**	-0,0189	0,283*
	**Significance**	<0,0001		0,0447	<0,0001	0,0138	0,1067	<0,0001	0,0009	0,0453	0,1020	0,0108	<0,0001	0,0793	0,7848	<0,0001	0,9114	0,0110
**APACHE II**	**Spearman's Correlation coefficient**	0,1908	,229*	1,0000	,675**	-0,1516	,245*	,426**	-0,1771	0,0332	-,255*	-0,0473	-0,1848	0,2647	-0,2230	0,3190	0,0534	0,282*
	**Significance**	0,0988	0,0447		<0,0001	0,1912	0,0332	<0,0001	0,2618	0,7758	0,0260	0,6848	0,1100	0,0531	0,1557	0,0510	0,7606	<0,0001
**SOFA score**	**Spearman's Correlation coefficient**	,378**	,491**	,675**	1,0000	-0,0049	,244*	,624**	-,345*	-0,1545	-0,1934	-,306**	-,248*	,284*	-0,0925	,525**	-0,2093	0,437*
	**Significance**	0,0008	<0,0001	<0,0001		0,9667	0,0349	<0,0001	0,0271	0,1858	0,0964	0,0075	0,0323	0,0392	0,5603	0,0007	0,2348	<0,0001
**C-reactive protein**	**Spearman's Correlation coefficient**	,292**	,276*	-0,1516	-0,0049	1,0000	0,0689	-0,1423	0,2319	-0,1148	-0,0882	-0,0337	-0,0093	-0,1001	,521**	0,2159	-0,1271	0,1870
	**Significance**	0,0094	0,0138	0,1912	0,9667		0,5464	0,2233	0,1253	0,3138	0,4394	0,7681	0,9352	0,4587	<0,0001	0,1809	0,4535	0,0980
**Procalcitonin**	**Spearman's Correlation coefficient**	,319**	0,1829	,245*	,244*	0,0689	1,0000	,380**	-,405**	0,0274	-0,0330	-,386**	-,311**	0,1933	-0,1220	,559**	-0,1569	-0,0260
	**Significance**	0,0044	0,1067	0,0332	0,0349	0,5464		0,0008	0,0058	0,8108	0,7727	0,0004	0,0052	0,1497	0,4302	<0,0001	0,3536	0,8180
**Lactate**	**Spearman's Correlation coefficient**	,410**	,509**	,426**	,624**	-0,1423	,380**	1,0000	-,371*	0,0496	-,242*	-0,1366	-,418**	0,2003	-0,2747	,359*	-,342*	0,245*
	**Significance**	<0,0001	<0,0001	<0,0001	<0,0001	0,2233	0,0008		0,0170	0,6723	0,0362	0,2425	<0,0001	0,1504	0,0783	0,0267	0,0478	0,0340
**Functional protein C**	**Spearman's Correlation coefficient**	-0,2719	-,477**	-0,1771	-,345*	0,2319	-,405**	-,371*	1,0000	,315*	0,1011	,524**	,538**	-,664**	,478*	-,523**	0,2317	-0,0690
	**Significance**	0,0708	0,0009	0,2618	0,0271	0,1253	0,0058	0,0170		0,0349	0,5089	<0,0001	<0,0001	<0,0001	0,0101	0,0073	0,2548	0,6550
**Leukocytes**	**Spearman's Correlation coefficient**	-0,0079	-,226*	0,0332	-0,1545	-0,1148	0,0274	0,0496	,315*	1,0000	-0,0888	,241*	-0,1332	0,0359	-0,0958	-0,0814	0,0972	-0,4200
	**Significance**	0,9455	0,0453	0,7758	0,1858	0,3138	0,8108	0,6723	0,0349		0,4363	0,0323	0,2420	0,7908	0,5363	0,6174	0,5670	0,7120
**Hemoglobin**	**Spearman's Correlation coefficient**	-0,2073	-0,1854	-,255*	-0,1934	-0,0882	-0,0330	-,242*	0,1011	-0,0888	1,0000	-0,0130	,328**	-,496**	0,0317	-0,2479	0,0367	-0,2060
	**Significance**	0,0686	0,1020	0,0260	0,0964	0,4394	0,7727	0,0362	0,5089	0,4363		0,9096	0,0032	<0,0001	0,8383	0,1230	0,8292	0,0690
**Platelets**	**Spearman's Correlation coefficient**	-,269*	-,285*	-0,0473	-,306**	-0,0337	-,386**	-0,1366	,524**	,241*	-0,0130	1,0000	,300**	-,274*	0,2598	-,564**	0,0956	-0,0220
	**Significance**	0,0171	0,0108	0,6848	0,0075	0,7681	<0,0001	0,2425	<0,0001	0,0323	0,9096		0,0072	0,0392	0,0885	<0,0001	0,5737	0,8490
**Quick index**	**Spearman's Correlation coefficient**	-,446**	-,411**	-0,1848	-,248*	-0,0093	-,311**	-,418**	,538**	-0,1332	,328**	,300**	1,0000	-,532**	,333*	-,545**	0,0507	-0,4600
	**Significance**	<0,0001	<0,0001	0,1100	0,0323	0,9352	0,0052	<0,0001	<0,0001	0,2420	0,0032	0,0072		<0,0001	0,0271	<0,0001	0,7659	0,6890
**Activated Partial Thromboplastin Time**	**Spearman's Correlation coefficient**	0,2399	0,2344	0,2647	,284*	-0,1001	0,1933	0,2003	-,664**	0,0359	-,496**	-,274*	-,532**	1,0000	-,446**	,395*	0,1062	0,2390
	**Significance**	0,0723	0,0793	0,0531	0,0392	0,4587	0,1497	0,1504	<0,0001	0,7908	<0,0001	0,0392	<0,0001		0,0039	0,0190	0,5565	0,0730
**Fibrinogen**	**Spearman's Correlation coefficient**	0,1322	0,0424	-0,2230	-0,0925	,521**	-0,1220	-0,2747	,478*	-0,0958	0,0317	0,2598	,333*	-,446**	1,0000	-0,1452	0,3513	0,2130
	**Significance**	0,3923	0,7848	0,1557	0,5603	<0,0001	0,4302	0,0783	0,0101	0,5363	0,8383	0,0885	0,0271	0,0039		0,4280	0,0723	0,1650
**Dimer-D**	**Spearman's Correlation coefficient**	,725**	,541**	0,3190	,525**	0,2159	,559**	,359*	-,523**	-0,0814	-0,2479	-,564**	-,545**	,395*	-0,1452	1,0000	-0,1973	0,0910
	**Significance**	<0,0001	<0,0001	0,0510	0,0007	0,1809	<0,0001	0,0267	0,0073	0,6174	0,1230	<0,0001	<0,0001	0,0190	0,4280		0,3788	0,5780
**Prothrombin time**	**Spearman's Correlation coefficient**	0,1198	-0,0189	0,0534	-0,2093	-0,1271	-0,1569	-,342*	0,2317	0,0972	0,0367	0,0956	0,0507	0,1062	0,3513	-0,1973	1,0000	0,1130
	**Significance**	0,4801	0,9114	0,7606	0,2348	0,4535	0,3536	0,0478	0,2548	0,5670	0,8292	0,5737	0,7659	0,5565	0,0723	0,3788		0,5040
**ICU Lenght of stay**	**Spearman's Correlation coefficient**	0,301*	0,283*	0,282*	0,437*	0,1870	-0,0260	0,245*	-0,0690	-0,0420	-0,2060	-0,0220	-0,0460	0,2390	0,2130	0,0910	0,1130	1,0000
	**Significance**	0,0070	0,0110	0,0140	<0,0001	0,0980	0,8180	0,0340	0,6550	0,7120	0,0690	0,8490	0,6890	0,0730	0,1650	0,5780	0,5040	

Table 2. Septic shock cases correlation analysis

Variable		H2B	H3	APACHE II	SOFA score	C-reactive protein	Procalcitonin	Lactate	Functional protein C	Leukocytes	Hemoglobin	Platelets	Quick index	Activated Partial Thromboplastin Time	Fibrinogen	Dimer-D	Prothrombin time	ICU Lenght of stay
H2B	Spearman's Correlation coefficient	1,0000	,812**	0,1709	,390**	,342**	,286*	,409**	-,320*	-0,0228	-0,2211	-,262*	-,485**	,284*	0,0936	,742**	0,1197	0,315*
	Significance		<0,0001	0,1735	0,0015	0,0046	0,0188	0,0008	0,0471	0,8547	0,0721	0,0320	<0,0001	0,0416	0,5657	<0,0001	0,4935	0,0090
H3	Spearman's Correlation coefficient	,812**	1,0000	0,1873	,466**	,329**	0,1028	,475**	-,425**	-0,2195	-0,1864	-,241*	-,385**	0,2141	0,0808	,519**	0,0579	0,298*
	Significance	<0,0001		0,1353	0,0001	0,0065	0,4078	0,0001	0,0069	0,0743	0,1309	0,0496	0,0013	0,1274	0,6200	0,0012	0,7413	0,0140
APACHE II	Spearman's Correlation coefficient	0,1709	0,1873	1,0000	,643**	-0,1315	0,1436	,341**	-0,0829	-0,0125	-0,2139	-0,0622	-0,1024	0,2626	-0,2133	0,2810	0,1880	0,305*
	Significance	0,1735	0,1353		<0,0001	0,3005	0,2575	0,0059	0,6309	0,9217	0,0896	0,6253	0,4206	0,0683	0,1986	0,1075	0,2949	0,0130
SOFA score	Spearman's Correlation coefficient	,390**	,466**	,643**	1,0000	-0,0084	0,0333	,567**	-0,2434	-0,1505	-0,1409	-,310*	-0,1451	0,2590	-0,1022	,465**	-0,1400	0,468*
	Significance	0,0015	0,0001	<0,0001		0,9482	0,7956	<0,0001	0,1589	0,2391	0,2708	0,0133	0,2565	0,0755	0,5414	0,0055	0,4447	<0,0001
C-reactive protein	Spearman's Correlation coefficient	,342**	,329**	-0,1315	-0,0084	1,0000	0,0699	-0,1381	0,1784	-0,0891	-0,0449	-0,0599	-0,0065	-0,1039	,519**	0,2337	-0,1743	0,1590
	Significance	0,0046	0,0065	0,3005	0,9482		0,5742	0,2803	0,2772	0,4733	0,7180	0,6302	0,9581	0,4634	0,0006	0,1701	0,3165	0,1960
Procalcitonin	Spearman's Correlation coefficient	,286*	0,1028	0,1436	0,0333	0,0699	1,0000	0,2319	-,370*	0,0755	0,0021	-,372**	-0,2035	0,1399	-0,0892	,536**	-0,0841	-0,0490
	Significance	0,0188	0,4078	0,2575	0,7956	0,5742		0,0675	0,0202	0,5436	0,9867	0,0019	0,0986	0,3224	0,5842	0,0007	0,6309	0,6930
Lactate	Spearman's Correlation coefficient	,409**	,475**	,341**	,567**	-0,1381	0,2319	1,0000	-0,2628	0,0608	-0,2398	-0,0803	-,338**	0,1548	-0,2570	0,2784	-0,2911	0,311*
	Significance	0,0008	0,0001	0,0059	0,0000	0,2803	0,0675		0,1272	0,6360	0,0583	0,5318	0,0067	0,2934	0,1193	0,1109	0,1061	0,0120
Functional protein C	Spearman's Correlation coefficient	-,320*	-,425**	-0,0829	-0,2434	0,1784	-,370*	-0,2628	1,0000	0,2948	0,0766	,521**	,523**	-,654**	,475*	-,481*	0,1578	-0,0970
	Significance	0,0471	0,0069	0,6309	0,1589	0,2772	0,0202	0,1272		0,0685	0,6432	0,0007	0,0006	0,0001	0,0190	0,0233	0,4512	0,5510
Leukocytes	Spearman's Correlation coefficient	-0,0228	-0,2195	-0,0125	-0,1505	-0,0891	0,0755	0,0608	0,2948	1,0000	-0,1278	0,2169	-0,1177	0,1167	-0,0697	-0,0790	0,1281	0,0140
	Significance	0,8547	0,0743	0,9217	0,2391	0,4733	0,5436	0,6360	0,0685		0,3026	0,0779	0,3427	0,4101	0,6691	0,6468	0,4634	0,9090
Hemoglobin	Spearman's Correlation coefficient	-0,2211	-0,1864	-0,2139	-0,1409	-0,0449	0,0021	-0,2398	0,0766	-0,1278	1,0000	-0,0026	,314**	-,522**	0,0993	-0,2105	0,0230	-0,1480
	Significance	0,0721	0,1309	0,0896	0,2708	0,7180	0,9867	0,0583	0,6432	0,3026		0,9834	0,0097	0,0001	0,5420	0,2178	0,8959	0,2300
Platelets	Spearman's Correlation coefficient	-,262*	-,241*	-0,0622	-,310*	-0,0599	-,372**	-0,0803	,521**	0,2169	-0,0026	1,0000	,290*	-0,2491	0,1855	-,561**	-0,0020	0,0020
	Significance	0,0320	0,0496	0,6253	0,0133	0,6302	0,0019	0,5318	0,0007	0,0779	0,9834		0,0173	0,0750	0,2519	0,0004	0,9911	0,9860
Quick index	Spearman's Correlation coefficient	-,485**	-,385**	-0,1024	-0,1451	-0,0065	-0,2035	-,338**	,523**	-0,1177	,314**	,290*	1,0000	-,533**	,314*	-,532**	-0,0461	-0,0520
	Significance	<0,0001	0,0013	0,4206	0,2565	0,9581	0,0986	0,0067	0,0006	0,3427	0,0097	0,0173		0,0000	0,0488	0,0008	0,7925	0,6750
Activated Partial Thromboplastin Time	Spearman's Correlation coefficient	,284*	0,2141	0,2626	0,2590	-0,1039	0,1399	0,1548	-,654**	0,1167	-,522**	-0,2491	-,533**	1,0000	-,440**	,376*	0,1424	0,284*
	Significance	0,0416	0,1274	0,0683	0,0755	0,4634	0,3224	0,2934	0,0001	0,4101	0,0001	0,0750	0,0000		0,0072	0,0310	0,4369	0,0410
Fibrinogen	Spearman's Correlation coefficient	0,0936	0,0808	-0,2133	-0,1022	,519**	-0,0892	-0,2570	,475*	-0,0697	0,0993	0,1855	,314*	-,440**	1,0000	-0,1012	0,2987	0,1460
	Significance	0,5657	0,6200	0,1986	0,5414	0,0006	0,5842	0,1193	0,0190	0,6691	0,5420	0,2519	0,0488	0,0072		0,5946	0,1383	0,3690
Dimer-D	Spearman's Correlation coefficient	,742**	,519**	0,2810	,465**	0,2337	,536**	0,2784	-,481*	-0,0790	-0,2105	-,561**	-,532**	,376*	-0,1012	1,0000	-0,1738	0,0990
	Significance	0,0000	0,0012	0,1075	0,0055	0,1701	0,0007	0,1109	0,0233	0,6468	0,2178	0,0004	0,0008	0,0310	0,5946		0,4511	0,5640
Prothrombin time	Spearman's Correlation coefficient	0,1197	0,0579	0,1880	-0,1400	-0,1743	-0,0841	-0,2911	0,1578	0,1281	0,0230	-0,0020	-0,0461	0,1424	0,2987	-0,1738	1,0000	0,1920
	Significance	0,4935	0,7413	0,2949	0,4447	0,3165	0,6309	0,1061	0,4512	0,4634	0,8959	0,9911	0,7925	0,4369	0,1383	0,4511		0,2690
ICU Lenght of stay	Spearman's Correlation coefficient	0,315*	0,298*	0,305*	0,468*	0,1590	-0,0490	0,311*	-0,0970	0,0140	-0,1480	0,0020	-0,0520	0,284*	0,1460	0,0990	0,1920	1,0000
	Significance	0,0090	0,0140	0,0130	<0,0001	0,1960	0,6930	0,0120	0,5510	0,9090	0,2300	0,9860	0,6750	0,0410	0,3690	0,5640	0,2690	
